# Point-of-Care Ultrasound to Diagnose a Simple Ranula

**DOI:** 10.5811/westjem.2016.9.30890

**Published:** 2016-11-02

**Authors:** Ili Margalit, Ron Berant

**Affiliations:** Schneider Children’s Medical Center of Israel, Department of Pediatric Emergency Medicine, Petah Tikva, Israel

## Abstract

In the following vignette we demonstrate the use of point-of-care ultrasound to diagnose a simple ranula.

## CASE

**Figure 1:**
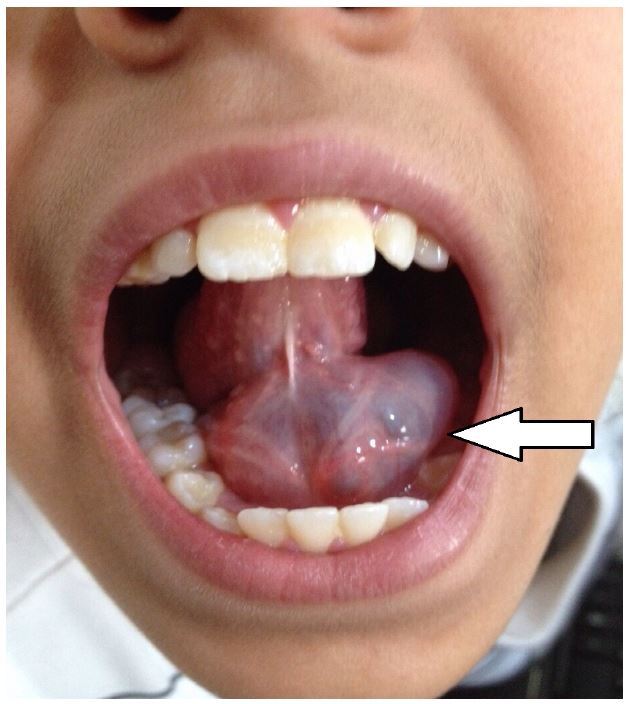
The sublingual mass, a simple ranula, seen on physical exam of a pediatric patient.

**Figure 2:**
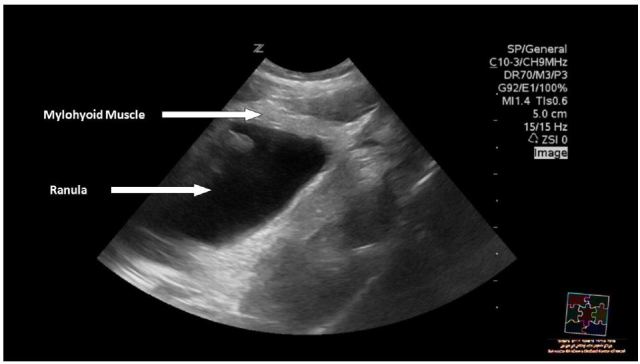
The ultrasonographic image, demonstrating the isolated ranula without extravasation through the mylohyoid muscle.

An 11-year-old previously healthy girl presented to the emergency department (ED) with three weeks of a rapidly progressive swelling underneath her tongue, causing difficulty in talking and eating. Physical examination revealed a 4.5 × 3 cm sublingual mass arising from the base of the tongue, around the midline (Figure 1). The mass was soft, movable and non-tender. The contents had a bluish hue, which was covered with normal appearing mucosa. A point-of-care ultrasound (POCUS) revealed a well-circumscribed homogenous cystic mass, separated from the muscular fibers of the tongue, without extravasation towards the neck (Figure 2) and without intra-cystic flow. A diagnosis of simple ranula was made.

## DISCUSSION

A ranula is a pseudocyst that is formed after oral trauma or inflammation, causing extravasation of mucous from the sublingual salivary gland or from the main salivary duct. A simple ranula is restricted to the oral cavity floor. A plunging ranula extravasates through the mylohyoid muscle, towards the cervical structures in the submandibular space.^1^ The differential diagnosis includes dermoid and epidermoid cysts as well as rarer conditions.^2^ Ultrasonography is a useful imaging method for the sublingual space, particularly for simple ranulas, as it is unaffected by dental amalgam and can locate the lesion.^3^ Furthermore, ultrasonography has been suggested as a key component in the management of floor-of-the-mouth masses in children.^4^ The now-accepted treatment of simple ranulas in pediatric patients consists of a six-month period of observation before considering other treatments.^1^ In this case, a POCUS was consistent with the clinical diagnosis, reassured the parents and prevented an additional medical visit as the entire management took place in the ED. The follow-up visit at the otorhinolaryngology clinic was scheduled for a few months later; by that time the ranula had completely resolved.
